# Characterization of Korean Distilled Liquor, *Soju*, Using Chemical, HS-SPME-GC-MS, and Sensory Descriptive Analysis

**DOI:** 10.3390/molecules27082429

**Published:** 2022-04-09

**Authors:** Hyoung-Uk Choi, Tae-Wan Kim, Seung-Joo Lee

**Affiliations:** 1 Department of Culinary and Food Service Management, Sejong University, Neungdong-ro 209, Gwangjin-gu, Seoul 05006, Korea; chlguddnr95@naver.com; 2 Traditional Foods Division, Korea Food Research Institute, Nongsangmyong-ro 245, Iseo-myeon, Wanju-gun 55365, Korea; ktwco@kfri.re.kr

**Keywords:** liquor, *soju*, GC-MS, SPME, volatile compounds, descriptive analysis, multiple factor analysis

## Abstract

The volatile compounds and sensory profiles of 18 different types of distilled *soju*, chosen with regard to various raw materials and distillation methods (atmospheric vs. vacuum), were explored using headspace solid-phase microextraction (HS-SPME) with gas chromatography-mass spectrometry (GC-MS) and descriptive analysis. General chemical properties such as pH, total acidity (TA), total soluble solids (°Brix), and lactic acid concentration were also determined. A total of 56 volatile compounds, comprising 31 esters, 11 alcohols, 1 acid, 4 aldehydes, 3 ketones, and 6 miscellaneous compounds, were identified. From the principal component analysis (PCA) of the volatile data, samples made using atmospheric distillation such as MSO and PJU showed a clear difference from decompressed distillation samples. Based on the PCA of the sensory data, there was also a clear distinction between samples by their distillation method. To explore relationships among chemical, volatile, and sensory data sets, multiple factor analysis (MFA) was applied. Yeasty and earthy flavors showed a close relationship with 1-nonanol, octatonic acid, and longer-chain esters such as ethyl phenylacetate and ethyl tetradecanoate, and with chemical parameters such as TA, °Brix, and lactic acid.

## 1. Introduction

*Soju* is one of the most popular alcoholic beverages in Korea. *Soju* is a distilled liquor manufactured using a pot still or continuous distillation after saccharification of grains or starchy raw materials [1]. Traditionally, distilled *soju* is made through a pot still at atmospheric pressure, but currently, modern vacuum distillation is widely applied and gaining more popularity in the industry. The vacuum distillation method, which is a method of distilling by lowering the pressure without applying direct heat by using a stainless-steel concentrator, has the advantage of maintaining high volatile aroma components with less heated and burnt flavors of the distilled liquor [2].

The headspace solid-phase microextraction (HS-SPME) methodology, which has the advantage of shortening the extraction time and increasing the sensitivity by using a variety of fibers, is generally applied to volatile analysis in alcoholic beverages as well as general foods [3]. HS-SPME is widely used to detect volatile compounds in whiskey [4], brandy [5], Chinese *Baijiu* [6], and other alcoholic beverages. The distribution and chemical composition of aroma compounds result from the fermentation raw material, fermentation and distillation conditions, aging containers, and durations of distilled liquor [7].

Traditional *soju* manufactured by atmospheric distillation leaks a lot of middle–high-boiling components compared to vacuum distilled liquor [8]. It was reported that the higher the distillation temperature, the more the esterification reaction occurs during the distillation, resulting in longer-chain ester compounds [9]. *Soju* distilled under atmospheric pressure has heated, burnt, and grainy flavors with a strong bitter taste. These flavors are gradually decreasing as atmospheric distillation is being replaced by vacuum distillation [8]. In the case of *soju* manufactured from mashed products using *nuruk* (Korean *koji*), the contents of ethyl nonanoate, ethyl lactate, 2-phenyl ethanol, and acetaldehyde were higher by more than two times in *soju* made under atmospheric distillation than in that made under vacuum distillation [7].

Distilled *soju* uses various types of raw materials such as glutinous rice, barley, non-glutinous rice, wheat, sorghum, and corn. Various types of longer-chain esters such as ethyl decanoate, ethyl dodecanoate, ethyl tetradecanoate, ethyl hexadecanoate, ethyl 9-octadecenoate, and ethyl octadecanoate were major aroma ingredients in *soju* manufactured using sweet potatoes [10]. Commercial Japanese *shochu* did not show significant differences in aroma patterns according to the raw materials—rice and barley [11]. Ethyl decanoate, ethyl dodecanoate, ethyl octanoate, isoamyl alcohol, and isobutyl alcohol were major volatile components in *soju* made with potato [12]. The volatile components of distilled *soju* aging in different containers (oak, pottery, or stainless steel) over 18 months were investigated using HS-SPME [13]. *Soju* aged in an oak barrel was higher in aldehydes, ketones, and miscellaneous compounds than that in other aging containers [13]. In addition, in the case of oak barrel aging, furfural and oakactone were detected, differently from the *soju* aged in stainless steel, which are known to have burnt, oaky, woody, and coconut flavors [14]. These esters, alcohols, and other volatiles are widely found in distilled *soju*, beer, *shochu*, rice wine, etc., and have a great influence on the quality of liquors [15].

Depending on various manufacturing conditions, there is a big difference not only in aroma compounds but also in sensory characteristics [15]. In the study of seven commercial distilled *soju*, bitterness, bitter aftertaste, sake odor/flavor, corn silk odor, acetone odor, and soy sauce odor were determined as major sensory characteristics [16]. In addition, a sensory descriptive analysis of nine locally famous distilled *soju* was performed with more detailed sensory characteristics such as eight aroma, four flavor/taste, and six mouthfeel-related attributes [17]. Volatile compounds as well as chemical properties in distilled *soju* have a great influence on the quality and sensory characteristics of the product. Therefore, analysis of the main volatile compounds in chemical compositions of distilled *soju* is important for quality improvement and new product development.

To relate the instrumental data to sensory profiles, multivariate statistical methods are required. The ultimate goal is to apprehend how differences in sensory profiles are caused by variations in instrumental data. Several multivariate techniques have been applied in food and flavor research, as reviewed by Noble and Ebeler [18], and by Pages and Husson [19]. Among various multivariable techniques, multiple factor analysis (MFA) is useful to simultaneously analyze several tables of variables—chemical, volatile, and sensory profiles in this study—and to obtain results that allow studying the relationship among the observations, the variables, and the tables. MFA is widely applied in various food products such as bryndza cheese [20], orange juice [21], and coffees from different terroirs [22,23].

The objective of this research was to quantify and compare the general chemical composition, volatile compounds, and sensory characteristics of eighteen commercial distilled *soju*. *Soju* samples were analyzed by HS-SPME and GC-MS. Additionally, sensory descriptive analysis was applied to derive sensory characteristics. MFA was conducted to explore the relationships among different data sets such as chemical, volatile, and sensory data sets.

## 2. Materials and Methods

### 2.1. Materials and Chemicals

Various commercial distilled *soju* samples were screened using online markets, postal shopping services for traditional Korean liquors, or liquor wholesale stores. Initially, twenty-eight samples from different manufacturers were purchased from the above sources. The samples were informally evaluated using blind taste tests by experienced drinkers, who also described the sensory characteristics and identified defective samples. Defective samples were eliminated from further consideration and among samples that were extremely similar. Finally, twenty samples were selected for descriptive analysis and subsequent instrumental tests. However, two samples were discontinued during the sensory tastings. Eventually, eighteen samples with regard to various raw materials, distillation methods, or aging methods were used in this study. Detailed information about the samples is presented in Table 1. 2-Methyl-1-pentanol (internal standard), n-alkane standards (C9–C25), and sodium chloride were purchased from Sigma–Aldrich Chemical Co. (St. Louis, MO, USA) for HS-SPME and GC-MS. Sodium sulphate (Junsei, Tokyo, Japan) and HPLC-grade solvents were used (J.T. Baker, NJ, USA). Other reagents used were purchased from Sigma–Aldrich (St. Louis, MO, USA). All chemical standards and reagents were analytical grade with at least 97% purity.

### 2.2. General Chemical Analysis

The pH of the samples was measured with a pH meter (520A; Orion Research Inc., Franklin, MA, USA). The titratable acidity (acetic acid in grams per liter) was measured by adding 10 g of sample to 50 mL of deionized water and titrating with 0.1 mol/L sodium hydroxide to an endpoint of pH 8.3. Total soluble solids (ºBx) were measured using an ATAGO hand refractometer (model N-1E, ATAGO, Tokyo, Japan). The lactic acid concentration was analyzed using high-performance liquid chromatography (HPLC) procedures adapted from Lee et al. [24]. The HPLC (Waters UV-2487, Miliford, MA, USA) was equipped with a Bio-Rad HPLC Organic Acid Analysis Aminex HPX-87H ion exclusion column (300 × 7.8 mm). Lactic acids were detected at 210 nm using a UV detector. A calibration curve was prepared, and the results were expressed as mg L-lactic acids/L. All chemical measurements were repeated three times, and the average values were reported.

### 2.3. Volatile Compound Analysis

#### 2.3.1. Headspace Solid-Phase Microextraction and Gas Chromatography-Mass Spectrometry Analysis

To extract the diverse volatile compounds in distilled *soju* samples, different types of SPME fibers were considered such as polydimethylsiloxane (PDMS; 100 µm), carboxen/polydimethylsiloxane (CAR/PDMS; 85 µm), polymethylsiloxane/divinylbenzene (PDMS/DVB; 65 µm), and divinylbenzene/carboxen/PDMS (DCP, 50/30 µm) from Supelco (Bellefonte, PA, USA). The DCP (50/30 µm) SPME fiber was selected, which showed a superior extraction capability for the various volatiles. To minimize the interferences of ethyl alcohol during the extraction, the alcohol content of each sample was adjusted to 10% (*w*/*v*) using distilled water. The HS-SPME procedure was adapted from previous studies [13,25]. A diluted distilled *soju* sample (5 g) with 100 µL of the internal standard, 2-methyl-1-pentanol (100 ng/mL in distilled water), and 1 g of sodium chloride was placed in a 20 mL headspace glass vial (Supelco, Bellefonte, PA, USA). A conditioned SPME fiber was exposed to the headspace of the shaking sample at 40 °C for 30 min in an autosampler (combi PAL G6504-CTC; CTC Analytics, Zwingen, Switzerland). The fiber was then retracted and immediately injected into the gas chromatography (GC) inlet on a Hewlett-Packard (HP) gas chromatograph model 7890A (Palo Alto, CA, USA) coupled to an Agilent 5975C mass spectrometer (Agilent Technologies, Santa Clara, CA, USA) with a Stabilwax^®^-DA bonded fused capillary column (30 m × 0.25 mm i.d. × 0.25 μm film thickness; Restek, Bellefonte, PA, USA). The GC-MS operating conditions were described in previous studies [13,25].

#### 2.3.2. Identification and Quantitation of Volatile Compounds

Volatile compounds were tentatively identified by comparison of the Kovats retention index (KI) [26] and the MS fragmentation patterns with those of reference compounds or with mass spectra in the Wiley 275 mass spectral database (Hewlett-Packard, Palo Alto, CA, USA). The KI of unknown compounds was determined via injection with a homologous series of alkanes (C9–C25). The GC/MS conditions were the same as described in Section 2.3.1. The relative concentrations of the volatiles were determined by the averaged integrated areas based on the TIC from the duplicate run, normalized to the area of the internal standard (2-methyl-1-pentanol), assuming a response factor of 1.

### 2.4. Sensory Descriptive Analysis

For the descriptive analysis of eighteen distilled *soju* samples, six females and four males (age 25–42 years) were recruited based on interest and availability, as well as their liking of liquors, from Sejong University, Seoul, Korea. Seven 1.5 h training sessions were conducted, and consensus was reached for thirteen aroma, ten flavor/taste, and five mouthfeel attributes, as shown in Table 2. Standards were presented to deliberate each sensory attribute during the training and formal sessions. Five samples per session were evaluated, in duplicate, and a total of 8 sessions were conducted. The presentation order of each sample was randomized for each session by a Williams Latin square design [27]. To minimize the differences in alcohol levels among the presented samples, the alcohol level of the samples was adjusted to 20% *w*/*v* with distilled water. Then, 50 mL aliquots of samples were presented in clear plastic cups marked with three-digit numbers and covered with Petri dishes. The intensity of each attribute was evaluated on a scale of 0 to 9, where 9 was the highest possible score (i.e., most intense). Water and white bread were used to cleanse the palates of the panelists between samples [28]. All sensory testing was conducted in sensory booths at room temperature.

### 2.5. Statistical Analysis

Analyses of variance (ANOVAs) were run on the sensory descriptive data using SAS ver.6.12 (SAS Institute, Cary, NC, USA) by employing a three-way mixed model (judges, samples, and replications), with all two-way interactions with judges treated as random. Individual product differences were identified by Fisher’s least significant difference (LSD) test. The mean intensities of 22 significantly different sensory attributes were used to perform principal component analysis (PCA) using the covariance matrix with no rotation on XLSTAT ver. 2022.1 (Addinsoft, New York, NY, USA). PCA was also performed on the mean concentrations of 56 volatiles detected in more than 6 samples using the correlation matrix. These volatiles were significantly different among samples by two-way ANOVA (sample, duplicate injections). To evaluate any relationships among the chemical, volatile, and sensory data, multiple factor analysis (MFA) was conducted with XLSTAT using 4 chemical compositions, 22 sensory attributes, and 56 volatile compounds.

## 3. Results and Discussion

### 3.1. General Chemical Compositions of 18 Distilled Soju Samples

The general chemical compositions of the 18 distilled *soju* samples are presented in Table 3. While Korean traditional distilled *soju* samples have been reported to have an acidic pH level of 3.40–4.99 [29], the pH ranges of the 18 commercial samples in this study showed large differences from 3.62 to 7.42. The pH level of MSO using atmospheric distillation was the lowest at 3.62, while R25 using blends both from atmospheric and vacuum distillation, MIR, and JRO showed the highest levels of 7.42, 7.30, and 7.24, respectively. According to esterification studies of organic acids [1], JRO, MIR, and R25 seem to have the highest outflow of organic acids during distillation. The total acidity also showed a large difference of 0.02–0.31%. MSO and HBI had total outputs of 0.31% and 0.29%, which were much higher than those of other samples. HGO, JRO, MIR, and R25 had the lowest total output of 0.02%. Studies have shown that the acidity of alcoholic beverages greatly affects the flavor and preservation of products, and it is reported that the lower the acidity, the less perceived the pungent acidity of alcohols [2]. The total soluble solids had levels of 8.10–16.20°Brix, with the lowest level of 8.10 found in DJA made under vacuum distillation and the highest level of 16.20 found in CTO, which was a 15-month oak-aged sample. Through organic acid analysis, only lactic acid was detected in the 18 samples. No other organic acids were detected. The content of lactic acid was the highest in MSO at 1610.70 µg/mL, which was also the highest in total acidity. The organic acid contents of liquors fermented with traditional *nuruk* using wild microflora were higher than those using selected enzymes [8]. Accordingly, MSO made with traditional *nuruk* showed a high acidity due to the higher production of organic acids.

### 3.2. Compositions of Volatile Compounds

Primarily, a total of 120 volatile compounds were detected by GC-MS analysis of 18 distilled *soju* extracted with SPME fiber. Among them, 56 volatile compounds detected in more than 6 samples are listed in Table 4 according to their chemical classes, relative concentrations, and Kovats indices (KIs), including 31 esters, 11 alcohols, 1 acid, 4 aldehydes, 3 ketones, and 6 miscellaneous compounds. Esters and alcohols were the largest classes among the quantified volatiles. The two most abundant volatile compounds were ethyl octanoate and ethyl decanoate, accounting for 31.41% and 31.02% of the total quantified volatiles. In addition, ethyl dodecanoate (6.39%), ethyl hexanoate (4.62%), isoamyl acetate (2.15%), 1-pentanol (11.88%), and phenylethyl alcohol (2.28%) were major volatiles in 18 samples. These compounds were also major volatiles in various alcoholic beverages [5,20,21]. R40 (738.16 mg/L), which was atmospherically distilled using 100% sweet potato, retained the highest total quantified volatile compounds, followed by MSO (720.32 mg/L) made under atmospheric distillation of non-glutinous rice, and DJA (482.88 mg/L), which was vacuum distilled using rice.

A total of 31 esters were detected among the 18 samples. Esters have a big influence on the aroma characteristics of alcoholic beverages with strong fruit-related notes [30]. Ethyl octanoate described as a sweet and fruity aroma [31] and ethyl decanoate described as a floral and sweet odor [32] were the two most frequently detected compounds among the 18 samples, and in particular, MSO and R40, which were atmospherically distilled, showed much higher levels of these compounds. In addition, isoamyl acetate, 2-phenylethyl acetate, ethyl heptanoate, and diethyl butanedioate were identified as major compounds. Among the various esters detected in this study, isoamyl acetate with a banana-like flavor [33], ethyl acetate having a sweet and fruity hint [34], and 2-phenylethyl acetate described as a rose or apple-like odor [35] are considered potent fruity compounds, which are also found in various distilled *soju*, beers, *cheongju*, and rice wines [35,36,37,38].

Alcohols are major compounds that give various aroma characteristics to liquor along with esters [36]. In the case of alcohol compounds, unlike the esters, the differences among samples were not large. GSO (41.38 mg/L) made under atmospheric distillation showed the highest contents of alcohols, and MBA (20.04 mg/L) made under reduced pressure distillation using millet, sorghum, and rice showed the lowest. Isoamyl alcohol, isobutyl alcohol having a fruity flavor [35], and phenylethyl alcohol having a rose odor [37] were detected as major alcohol components. Isoamyl alcohol is known to give off a harmonious flavor when there is an appropriate amount in the liquor, but when there is a large amount, it has an unpleasant, musty flavor [38]. These alcohols are commonly found in various alcoholic beverages such as *soju*, wine, whiskey, and brandy [30,31,32,33,34,35,36,37,38]. Volatile acids are known to be inappropriate in alcoholic beverages [37]. Octanoic acid was detected in six samples.

**Figure 1:**
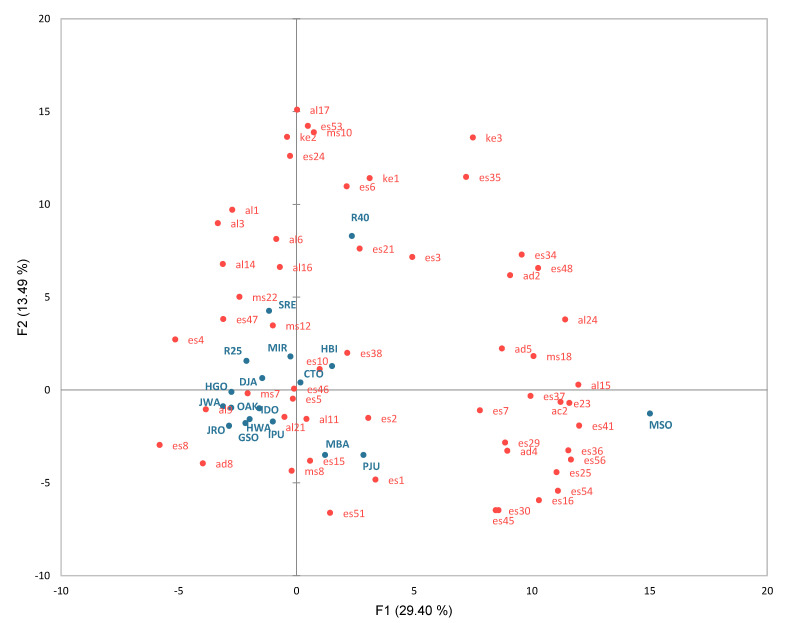
Principal component analysis loading for 56 volatile compounds (red) and scores for the 18 distilled *soju* samples (blue). The samples and volatile compound codes are defined in Table 3.

Principal component analysis (PCA) was performed using 56 volatile compounds detected in 6 or more samples to determine the overall distribution of volatile substances according to sample separation. The first principal component (PC1) showed 29.40% explanatory power, while the second principal component (PC2) showed 13.49%, as shown in Figure 1. The volatile compounds found on the positive side of PC1 (Figure 1) were ethyl lactate (es16), ethyl undecanoate (es41), ethyl dodecanoate (es48), ethyl tetradecanoate (es56), 1-nonanol (al15), octanoic acid (ac2), nonanal (ad2), and benzenaldehyde (ad5). These compounds are considered to be the major compounds of the atmospherically distilled samples rather than the vacuum distilled samples, as also determined in other studies [7,15]. Moreover, across the second principal component (PC2), there was a distinction among samples based on the major ingredients used for brewing. The sample brewed using only sweet potatoes (R40) was detected to have higher concentrations of esters such as ethyl 2-methylbutanoate (es6), propyl octanoate (es24), 2-methylbutyl octanoate (es35), and 2-decanone (ke2) along the positive side of PC2.

By examining the sample distribution in Figure 1, MSO, an atmospheric pressure distillation product, showed a significant difference along the positive side of PC1, compared to the other samples. PJU and R40 made under atmospheric distillation were also positioned on the positive side of PC1. Most of the other samples except MBA, HBI, and CTO were distributed on the negative side of PC1, which were vacuum distilled samples. Across PC2, there was a distinct separation between R40 and MBA, which were manufactured using sweet potatoes or various grains such as sorghum and millet, respectively. As a result, this plot demonstrates that the composition of volatile compounds in these samples differed according to the raw materials and the distillation method.

### 3.3. Sensory Characteristics of Distilled Soju Samples by Descriptive Analysis (DA)

To depict the sensory characteristics of the distilled *soju* samples, sensory descriptive analysis was performed. The mean intensity ratings of the 18 samples are presented in Table 5. The ANOVA performed on the 28 sensory attributes of the 18 samples revealed that all attributes except ‘metal’, ‘bitter’, ‘alcohol flavor’, ‘astringent’, ‘spicy’, and ‘swallow’ were significantly different across the samples (*p* < 0.05). Because the alcohol concentration of each sample was adjusted to the same level, a similar intensity of alcohol and mouthfeel-related sensory attributes could be expected. This outcome was also reported in the descriptive analysis of eleven distilled spirits also adjusted to the same alcohol level [15].

**Figure 2:**
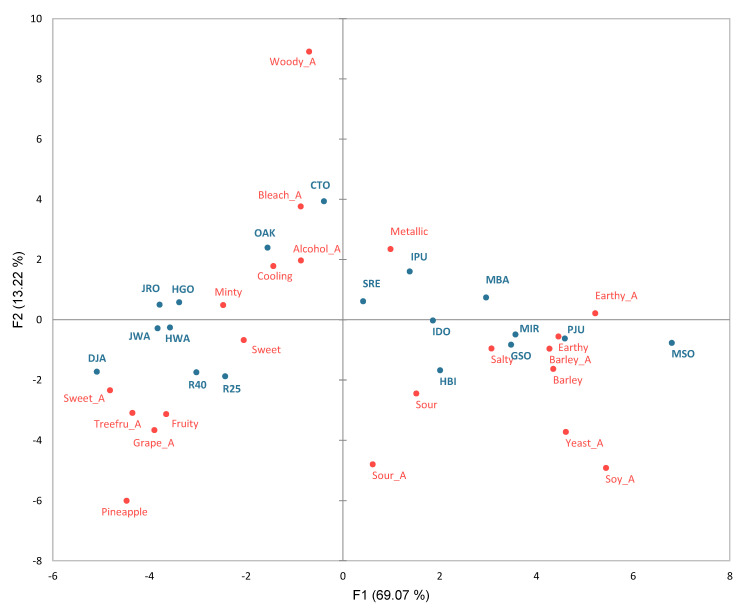
Principal component analysis loadings for 22 sensory attributes (red) and scores for the 18 distilled *soju* samples (blue). The samples and sensory attribute codes are defined in Table 1 and Table 2.

To examine the relationships among the sensory terms and separations of samples at a glance, principal component analysis (PCA) was performed using the mean ratings of 22 sensory characteristics showing significant differences across the 18 samples (Figure 2). PC1 and PC2 explained 69% and 13% of the variance across the samples, respectively. PC1 showed a contrast between ‘earthy’/‘yeasty’/‘barley’/‘soy_A’ and ‘sweet aroma’/‘fruity’/fruit-related aroma attributes, as shown in Figure 2. PC2 seemed to show a contrast between ‘woody_A’/‘bleach_A’ and ‘sour_A’. Examining the sample configuration (Figure 2), along PC1, there was a strong separation between samples shown by DA to be fruity and sweet and those which were low in fruitiness but high in sensory attributes related to yeasty and earthy. MSO, PJU, MIR, and GSO were located to the far right along PC1, indicating high levels in ‘earthy’, ‘yeasty’, ‘barley’, and ‘soy_A’. Likewise, these samples were made using atmospheric distillation, except for MIR. These typical sensory characteristics of atmospherically distilled liquors were also revealed in sensory studies of commercial distilled liquor products [15,17]. Conversely, DJA, R25, and R40 appeared on the negative side of PC1 with strong fruit- and sweet-related sensory characteristics. Along PC2, CTO and OAK, both aged in oak, were located on the far positive side, which showed significantly high intensities in ‘woody_A’ and ‘bleach_A’.

### 3.4. Relationships among Chemical, Volatile, and Sensory Profiles Using Multiple Factor Analysis

To understand the relationships among the chemical, volatile, and sensory data sets including 4 chemical compositions, 56 volatile compounds, and 22 sensory attributes, MFA was applied to the 18 distilled samples. From the global analysis of the MFA using the three data sets, the first two factorial axes accounted for 36.9% and 11.7% of the total variance, respectively. The first eigenvalue, 2.47, of the global MFA was very close to the maximum eigenvalue that could be reached, while the eigenvalue for the second factor was 0.78. In this sense, the first factor was a major direction for the interpretation of the MFA. The coordinates, respective contributions, and squared cosines of the groups of variables of the first two factors of the global MFA are presented in Table 6. The coordinates of the three data sets were highly related to the first factor as shown by the values of 0.84, 0.76, and 0.86 for the chemical, sensory, and volatile groups, respectively. The three data sets showed a similar contribution (30.9–35.1%) to the formation of F1, while volatile (42.5%) and sensory (25.6%) showed a difference in the formation of F2 (Table 3). The RV coefficient, which is a multivariate analogue of the correlation coefficient in MFA, was 0.59 between the chemical and volatile data, which is stronger than the RV coefficients of the chemical and sensory data or the volatile and sensory data, with values of 0.45 and 0.53, respectively.

**Figure 3:**
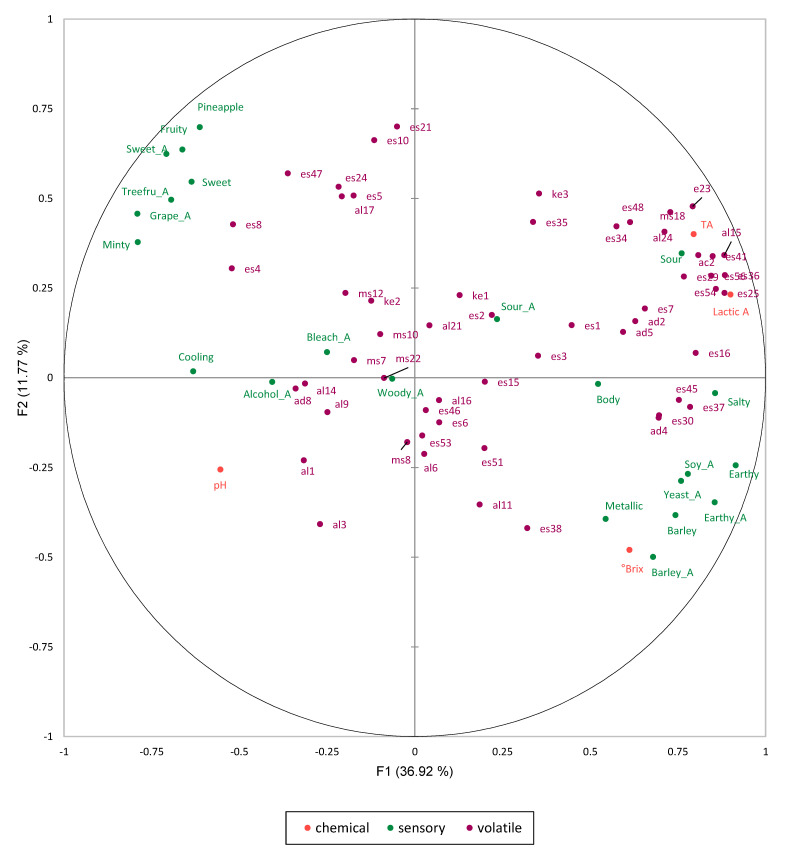
Representation of the projections of the active variables (chemical, sensory, and volatile data) for the MFA of the 18 distilled *soju* samples. The sample and chemical, volatile, and sensory attribute codes are defined in Table 1, Table 2, Table 3 and Table 4.

Figure 3 shows the projections of the active variables in the global MFA. The four chemical parameters, namely, lactic acid, TA, Brix, and pH, showed a strong contribution to F1 with values of 12.9, 10.1, 6.0, and 4.9%, respectively. Secondly, the sensory variables such as ‘earthy’, ‘yeasty’, ‘barley’, ‘soy_A’, ‘salty’, and ‘sour’ on the positive side of F1, with ‘sweet’, ‘fruity’, and fruit-related attributes on the opposite side, showed a strong contribution to the first factor. This distinction also appeared in the PCA of the sensory data (Figure 2). However, ‘woody_A’, ‘sour_A’, ‘bleach_A’, ‘body’, and ‘alcohol_A’ were found to show a weak contribution to F1 and F2. Because the volatile data consisted of 56 compounds, many compounds showed little contribution to the formation of factors 1 and 2, especially those positioned near the centroid. However, ethyl trans-4-decenoate (es36), ethyl nonanoate (es25), 1-nonanol (al15), ethyl 3-phenylpropanoate (es54), ethyl undecanoate (es41), ethyl tetradecanoate (es56), octanoic acid (ac2), ethyl lactate (es16), 3-methylbutyl octanoate (es23), diethyl succinate (es37), and ethyl phenylacetate (es45) showed a strong contribution to F1, with a clear association with the ‘earthy’, ‘yeasty’, ‘barley’, ‘soy_A’, ‘salty’, and ‘sour’ attributes. Furthermore, all of these compounds also showed strong positive correlations with TA and lactic acid, as well as with the ‘earthy’, ‘earthy_A’, ‘salty’, and ‘sour’ attributes (*p* < 0.05). Conversely, fruit- and sweet-related sensory attributes were highly correlated with isobutyl acetate (es4), isoamyl acetate (es8), and 2-phenylethyl acetate (es47) (*p* < 0.05).

**Figure 4:**
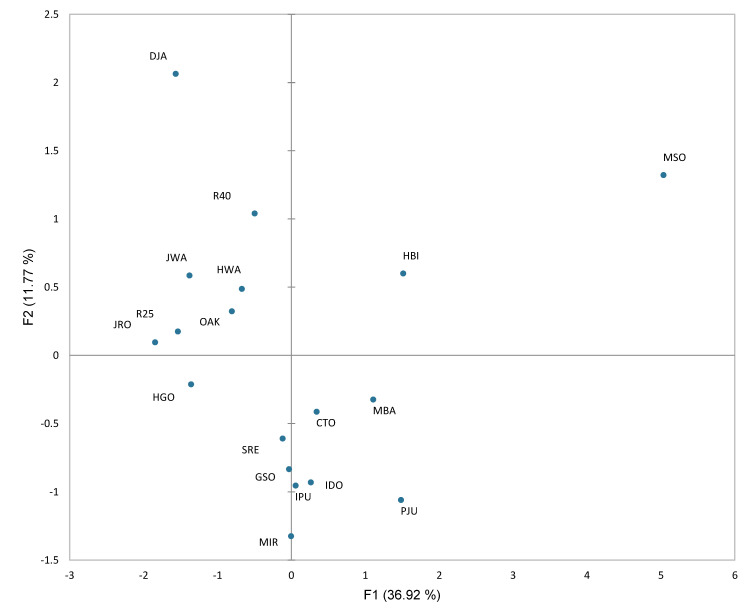
Projection of the 18 distilled *soju* samples by F1 and F2 of the global MFA using chemical, sensory, and volatile data. The sample codes are defined in Table 1.

The distribution of the 18 samples in the global MFA is presented in Figure 4. This plot shows an overall similar pattern to the sensory PCA. MSO, which showed prominent yeasty and earthy flavors under atmospheric pressure distillation, was located on the far-right side, with a dominant contribution (56.8%) to F1. Other atmospherically distilled samples such as PJU were also positioned on the right side of F1. However, atmospherically distilled GSO and R40 did not show a similar grouping. Other samples such as JRO, DJA, R25, and HBI showed higher levels of contributions to F1 in the range from 5.14 to 7.63%. CTO, IDO, SRE, IPU, GSO, and MIR showed a minimal contribution to F1. The DJA and R40 samples with strong fruit flavors and sweetness showed a higher contribution to F2, with 29.93 and 7.58%, respectively. The apparent grouping of the samples by their raw materials was not determined by MFA.

## 4. Conclusions

The volatile compounds and sensory characteristics of 18 distilled *soju* samples made with different types of materials and distillation methods were examined. The volatile composition of the samples was primarily determined by distillation methods (atmospheric vs. reduced pressure) and major raw ingredients such as sweet potato or rice. *Soju* samples manufactured using atmospheric distillation such as MSO and PJU had higher levels of longer-chain esters, 1-nonanol, and furfural, while the other samples produced by vacuum distillation showed much lower levels of those compounds. In addition, R40 made from sweet potatoes showed a distinctive sweet fruitiness in the volatile and sensory profiles compared to other samples made of grains and rice. As a result of sensory descriptive analysis, earthy and yeasty flavors with a barley hint were the main characteristics of the atmospherically distilled samples. From the MFA using chemical, sensory, and volatile data, the selected volatiles and chemical parameters showed strong interrelations with the sensory data. Volatiles such as 1-nonanol, octatonic acid, and longer-chain esters such as diethyl succinate, ethyl phenylacetate, and ethyl tetradecanoate and chemical parameters such as TA, °Brix, and lactic acid were highly associated with earthy and yeasty sensory characteristics, while isoamyl acetate, isobutyl acetate, and 2-phenylethyl acetate were related to sweet fruitiness. These components can be selected as preliminary sensory quality indicators of distilled *soju*, and it is expected that more accurate volatile compounds contributing to sensory characteristics can be identified through the analysis of trace volatile compounds.

## Figures and Tables

**Table 1:** Materials and their ingredients in distilled *soju* samples.

Code	Alcohol (%)	Raw Materials	Distillation Method	Aging Container
CTO	44.5	Rice	vacuum	Oak
DJA	21.0	Rice	vacuum	-
HBI	40.0	Rice	vacuum	Stainless
HGO	42.0	Rice	vacuum	Stainless
HWA	41.0	Rice	vacuum	Pottery
IDO	42.0	Rice	vacuum	Stainless
IPU	40.0	Rice	vacuum	Stainless
JRO	25.0	Rice	vacuum	Stainless
JWA	25.0	Rice, sweet potato	vacuum	Stainless
MBA	40.0	Rice, foxtail millet, sorghum	vacuum	Stainless
MIR	40.0	Rice	vacuum	Pottery
OAK	25.0	Rice	vacuum	Oak
SRE	40.0	Rice	vacuum	-
GSO	40.0	Rice, foxtail millet	atmospheric	Stainless
MSO	45.0	Non-glutinous rice	atmospheric	Stainless
PJU	42.0	Rice	atmospheric	Pottery
R25	25.0	Rice, sweet potato	atmospheric (sweet potato), vacuum (rice)	Pottery
R40	40.0	Sweet potato	atmospheric	Pottery

**Table 2:** Sensory codes, attributes, definitions, and physical standards of distilled *Soju*.

Attribute	Code	Written Definition	Physical Standards
***Aroma***			
Alcohol	Alcohol_A	Alcohol aroma	25% (*w*/*v*) ethanol
Sour_Aroma	Sour_A	Sour aroma	Acetic acid 2 mL/*distilled* water 100 mL
Sweet_Aroma	Sweet_A	Sweet aroma (e.g., honey/syrup)	Honey 20 mL/white sugar 20 g/*distilled* water 100 mL
Green Grape	Grape_A	Green grape aroma	Crushed green grape 30 g
Tree Fruit	Treefru_A	Tree fruit (e.g., pear/apple)	Apple juice, crushed Korean pear juice
Pineapple	Pineap_A	Pineapple aroma	Pineapple fruit bowl
Barley	Barley_A	Grain aroma (e.g., *nurungji*)	A typical barely drink
Yeast	Yeast_A	Activated yeast aroma (e.g., *Makgeolli*)	Yeast 0.1% in 10% warm sugar solution overnight
Earthy	Earthy_A	Wet basement, earthy aroma	Soil 30 g/*distilled* water 70 g
Soy Sauce	Soy_A	Soy sauce aroma (e.g., traditional Korean soy sauce)	Traditional Korean soy sauce 30 mL
Woody	Woody_A	Brandy aroma (e.g., wood, straw, fallen leaves)	70 mL brandy/30 mL distilled water
Metal	Metal_A	Metallic aroma	Stainless-steel sponge 30 g/tap water 20 g
Bleach	Bleach_A	Bleach aroma in hydrogen peroxide	2 mL hydrogen peroxide/100 mL distilled water
***Flavor*/*Taste***			
Bitter	Bitter	Bitter	Caffeine acid 0.1% (*w*/*v*)
Sour	Sour	Sour	Acetic acid 0.1% (*w*/*v*)
Salty	Salty	Salty	Salt 2 g/100 mL distilled water
Sweet	Sweet	Sweet	White sugar 20 g/distilled water 100 mL
Alcohol Flavor	Alcohol	Alcohol flavor	25% (*w*/*v*) ethanol
Fruit_Flavor	Fruity	Overall fruity flavor	Green grape 30 g, pineapple fruit bowl
Mint_Flavor	Minty	Mint flavor (e.g., Listerine)	Listerine 20 mL/distilled water 100 mL
Barley_Flavor	Barley	Savory flavor of grain	A typical barely drink
Earthy_Flavor	Earthy	Wet basement, earthy flavor	Soil 30 g/distilled water 70 g
Metal_Flavor	Metallic	Metallic flavor (e.g., iron, corroded iron)	Stainless-steel sponge 30 g/tap water 20 g
***Texture*/*Mouthfeel***			
Astringent	Astrin	Mouthfeel of dryness	Aluminum sulfate 0.1% (*w*/*v*)
Cooling Sensation	Cooling	Cool feeling	Peppermint candy 30 g
Spicy	Spicy	Feeling of stinging taste	Mustard flour 30 g in distilled water 15 mL
Swallow	Soft	Smooth swallowing from mouth to esophagus	No physical standards
Body	Body	Thickness or pressure of drinks on tongue, strong full mouthfeel	No physical standards

**Table 3:** General compositions of 18 distilled *soju* samples.

Sample Code	pH	Total Acidity (as Acetic Acid, % *v*/*w*)	Total Soluble Solids (°Brix)	Lactic Acids (mg/L)
CTO	4.33 ± 0.03 ^i^	0.09 ± 0.01 ^de^	16.20 ± 0.00 ^a^	375.39 ± 0.58 ^f^
DJA	4.84 ± 0.06 ^f^	0.08 ± 0.00 ^f^	8.10 ± 0.00 ^k^	8.28 ± 0.36 ^p^
GSO	5.60 ± 0.02 ^d^	0.04 ± 0.00 ^i^	14.70 ± 0.00 ^f^	114.39 ± 1.19 ^i^
HBI	3.90 ± 0.04 ^k^	0.29 ± 0.06 ^b^	14.70 ± 0.00 ^f^	435.06 ± 2.37 ^d^
HGO	6.31 ± 0.14 ^c^	0.02 ± 0.00 ^j^	15.20 ± 0.00 ^c^	66.44 ± 1.84 ^k^
HWA	4.65 ± 0.03 ^g^	0.08 ± 0.02 ^f^	14.90 ± 0.00 ^e^	486.02 ± 2.58 ^c^
IDO	4.48 ± 0.02 ^h^	0.09 ± 0.02 ^de^	15.20 ± 0.00 ^c^	156.09 ± 1.99 ^g^
IPU	4.82 ± 0.21 ^f^	0.06 ± 0.01 ^g^	14.90 ± 0.00 ^e^	53.86 ± 2.37 ^l^
JRO	7.24 ± 0.06 ^b^	0.02 ± 0.00 ^j^	9.73 ± 0.06 ^j^	41.40 ± 0.71 ^n^
JWA	4.33 ± 0.02 ^i^	0.09 ± 0.02 ^de^	9.70 ± 0.00 ^j^	35.40 ± 0.38 ^o^
MBA	4.27 ± 0.06 ^i^	0.10 ± 0.03 ^c^	14.67 ± 0.06 ^f^	590.88 ± 1.10 ^b^
MIR	7.30 ± 0.05 ^ab^	0.02 ± 0.00 ^j^	14.90 ± 0.00 ^e^	108.48 ± 0.31 ^j^
MSO	3.62 ± 0.02 ^l^	0.31 ± 0.06 ^a^	15.93 ± 0.06 ^b^	1610.70 ± 5.86 ^a^
OAK	4.04 ± 0.05 ^j^	0.09 ± 0.01 ^d^	10.27 ± 0.06 ^h^	117.10 ± 0.21 ^h^
PJU	4.38 ± 0.07 ^hi^	0.09 ± 0.02 ^d^	15.03 ± 0.06 ^d^	397.35 ± 2.45 ^e^
R25	7.42 ± 0.02 ^a^	0.02 ± 0.00 ^j^	9.90 ± 0.00 ^i^	44.50 ± 0.23 ^m^
R40	5.05 ± 0.03 ^e^	0.04 ± 0.00 ^hi^	14.70 ± 0.00 ^f^	47.61 ± 0.38 ^m^
SRE	4.92 ± 0.08 ^f^	0.05 ± 0.01 ^gh^	14.50 ± 0.00 ^g^	275.82 ± 3.45 ^e^
F-value	809.31 ***	484.41 ***	21,937.18 ***	115,098.93 ***

*** *p* < 0.001. ^a^^–p^ Different letters are significantly different at the 5% significance level by Duncan’s multiple range test.

**Table 4:** Volatile compounds (mg/L) ^(1)^ in 18 distilled *soju* samples.

Code	RI	KI ^(2)^	Volatile Compound ^(3)^	Samples ^(4)^																		Id ^(5)^
				CTO	DJA	GSO	HBI	HGO	HWA	IDO	IPU	JRO	JWA	MBA	MIR	MSO	OAK	PJU	R25	R40	SRE	
Esters																						
es1	896	887	Ethyl acetate	2.15	2.85	3.07	4.71		1.86	0.91	1.00		1.19	3.44		1.76	2.15	3.64		1.25	0.58	A
es2	970	966	n-Ethyl propanoate	0.12	0.07	0.09	0.07	0.09				0.06			0.06	0.10	0.06					A
es3	978	975	Ethyl 2-methylpropanoate	0.07			0.17		0.06						0.20	0.11	0.06	0.06	0.10	0.11		A
es4	1025	1018	Isobutyl acetate		0.10	0.14	0.10	0.20	0.12			0.08	0.09		0.06		0.08		0.06	0.10		A
es5	1049	1057	Ethyl butanoate	0.38	0.64	0.45	0.35	0.38	0.36		0.12	0.36	0.39	0.15	0.43	0.35	0.40	0.26	0.19	0.32		A
es6	1064	820	Ethyl 2-methylbutanoate	0.06			0.15		0.06		0.09				0.45	0.11	0.05	0.07	0.27	0.28	0.12	B
es7	1079	1075	Ethyl isovalerate	0.30			0.18		0.07		0.05					0.26	0.09	0.10		0.04		A
es8	1135	1130	Isoamyl acetate	2.26	9.54	6.24	5.04	8.37	12.80	1.70	2.61	6.78	7.19	4.85	1.65	0.80	6.25	1.01	1.88	3.44	2.75	A
es10	1247	1236	Ethyl hexanoate	2.65	74.17	2.22	9.38	4.31	6.00	1.78	7.20	10.29	3.46	10.84	5.27	16.84	3.22	3.57	8.55	8.92	4.01	A
es15	1347	1332	Ethyl heptanoate	0.11	0.53	0.04	1.53	0.26	0.76	0.12	1.18	0.40	0.17	1.44	0.31	0.18	0.20	0.66	0.28	0.27	0.47	A
es16	1356	1325	Ethyl lactate				0.77	0.12		0.09	0.59			0.47		5.64		4.97				A
es21	1449	1458	Ethyl octanoate	22.24	321.91	4.51	58.47	45.70	63.54	10.88	25.39	33.33	6.35	112.75	65.48	110.29	9.13	14.50	79.44	194.46	64.26	A
es23	1474	1452	3-Methylbutyl octanoate		1.57		1.01	0.14	0.42	0.16				1.40	0.74	4.93		1.02	0.30	0.96	0.33	A
es24	1534	1526	Propyl octanoate		0.39		0.18	0.10	0.06					0.08	0.12				0.06	0.33	0.18	A
es25	1550	1548	Ethyl nonanoate	0.18	0.49	0.06	3.97	0.25	1.35	0.24	0.75	0.04		7.05	1.69	13.18	0.04	2.40			1.51	A
es29	1574	1557	Ethyl (2E)-2-octenoate		0.11		1.02	0.04	0.22	0.07	0.20	0.04		0.91	0.18	1.24	0.05		0.10		0.22	A
es30	1586	1082	Isoamyl lactate				0.14	0.05		0.07	0.60			0.06		1.08		1.35			0.10	B
es34	1652	1652	Ethyl decanoate	5.46	22.36	0.97	55.87	6.23	46.24	7.70	0.19	0.63	0.10	189.00	44.07	367.16	0.34	27.46	12.62	380.08	60.88	A
es35	1673	1656	2-Methylbutyl octanoate				0.99	0.06	0.42						0.68	4.74				8.60		A
es36	1679	1698	Ethyl trans-4-decenoate				1.48							1.81	0.72	5.82		0.60			0.15	A
es37	1690	1694	Diethyl butanedioate	1.32	0.06	0.28	0.81	0.19	0.29	2.07	3.87	0.19	0.06	0.80	1.08	5.62	0.30	3.12	0.28	2.18	1.85	A
es38	1705	1683	Ethyl benzoate	0.17		0.03	0.36			0.05				0.47	0.46			0.63		0.22	0.45	A
es41	1754	1577	Ethyl undecanoate				0.19		0.02					0.29		2.51		0.55		0.35		B
es45	1818	1763	Ethyl phenylacetate	0.40	0.09		0.18		0.09	0.09	0.22	0.04	0.04	0.14	0.15	0.43	0.05	0.38				A
es46	1830	1804	Methyl salicylate	8.29	0.42				0.06		0.22	0.21	0.21	0.67			0.22		0.24			A
es47	1852	1826	2-Phenylethyl acetate	1.04	16.63	4.13	3.99	2.70	7.51	3.97	0.61	2.26	2.84				1.67	0.82	0.56	4.49	4.89	A
es48	1857	1846	Ethyl dodecanoate	0.38		0.21	5.93	0.21	9.07	1.62	0.07	0.06		22.74	1.73	100.34		12.76		88.97	8.54	A
es51	1894	955	2-Methyl-,1-(1,1-dimethylethyl)-2-methylpropanoic acid,-1,3-propanediyl ester		0.15				0.07					0.54			0.06	0.64	0.11		0.18	B
es53	1918	1894	Ethyl 3-methylbutyl succinate	0.16			0.12				0.15				0.17					0.29	0.34	A
es54	1923	1390	Ethyl 3-phenylpropanoate				0.12				0.04			0.47		0.90		0.17				B
es56	2060	2056	Ethyl tetradecanoate	0.39	0.65	0.10	1.41	0.12	4.92	0.51		0.10		4.32	0.29	33.56		11.61	0.09	2.29	1.45	A
Total esters				48.13	452.73	22.54	158.69	69.52	156.37	32.03	45.15	54.87	22.09	364.69	125.99	677.95	24.42	92.35	105.13	697.95	153.26	
Alcohols																						
al1	1097	1114	Iso-butyl alcohol	0.61	0.53	0.80	0.65	1.28	0.66	0.99	0.68	0.69	0.74	0.25	1.04	0.64	0.78	0.74	1.04	1.10	0.92	A
al3	1213	1261	Iso-amyl alcohol	25.31	17.74	27.07	24.35	34.88	21.65	30.50	20.84	27.28	25.37	16.35	32.17	21.53	27.14	25.65	27.13	30.15	34.88	A
al6	1359	1359	1-Hexanol	0.19			0.29	0.05				0.07			0.19				0.07		0.68	A
al9	1494	1019	2-Ethylhexanol	0.33	0.21	0.23		0.47		0.28	0.37	0.08			0.33		1.66		0.24		0.19	B
al11	1522	1511	2-Nonanol	0.15	0.11		0.11	0.11		0.12	0.09			0.08				0.35			0.26	A
al14	1637	1589	Ethanol, 2-(2-ethoxyethoxy)-		0.03				0.04				0.04		0.06				0.07		0.09	A
al15	1666	1661	1-Nonanol	0.32	0.21	0.08	0.63	0.26	0.08	0.16	0.19	0.06	0.11	0.38	0.18	1.82	0.13	0.44	0.17	0.38	0.42	A
al16	1769	1783	1-Decanol	0.33			0.43	0.16									0.05		0.10		0.44	A
al17	1773	1770	Citronellol		0.38	0.04	0.23	0.15		0.07							0.08		0.10	0.65	0.24	A
al21	1947	1931	Phenylethyl alcohol	9.99	8.41	12.98	9.14			5.73	1.75	6.58	6.35	2.98	2.63	4.48	6.61	3.77	2.76	3.98	2.20	A
al24	2047	1564	(6E)-3,7,11-Trimethyl-1,6,10-dodecatrien-3-ol				0.06								0.13	1.36		0.17	0.14	0.62		B
Total alcohols				37.23	27.62	41.20	35.89	37.36	22.43	37.85	23.92	34.76	32.61	20.04	36.73	29.83	36.45	31.12	31.82	36.88	40.32	
Acids																						
ac2	2073	2070	Octanoic acid	0.94			0.36			0.15				0.16		2.68				0.26		A
Total acids				0.94			0.36			0.15				0.16		2.68				0.26		
Aldehydes																						
ad2	1415	1382	Nonanal	0.22			0.23	0.34		0.31						0.70		0.41		0.55	0.32	A
ad4	1491	1483	Furfural	0.22		2.42					0.11				2.92	4.88	0.56	3.65	0.16	0.66		A
ad5	1566	1529	Benzaldehyde	0.48	0.73	0.26				0.42		0.18	0.19		2.58	3.67	0.24		0.26	0.22	1.07	A
ad8	1757	1195	Benzaldehyde, 3-ethyl-								0.04	0.08	0.05	0.07			0.06		0.06		0.04	B
Total aldehydes				0.92	0.73	2.68	0.23	0.34	0.00	0.73	0.15	0.26	0.24	0.07	5.50	9.25	0.86	4.06	0.48	1.43	1.43	
Ketones																						
ke1	1408	1397	2-Nonanone	0.12	0.08		0.07				0.06				0.04	0.07			0.11	0.08	0.11	A
ke2	1514	1493	2-Decanone	0.10			0.06						0.05				0.06		0.06	0.13	0.07	A
ke3	1620	1595	2-Undecanone	0.13	0.23		0.23							0.13	0.13	0.54			0.25	0.76	0.32	A
Total ketons				0.35	0.31	0.00	0.36	0.00	0.00	0.00	0.06	0.00	0.05	0.13	0.17	0.61	0.06	0.00	0.42	0.97	0.50	
Miscellaneous																						
ms7	1156	1142	p-Xylene	0.12	0.10	0.07			0.28		0.05			0.05	0.08		0.05	0.08	0.07	0.07	0.04	A
ms8	1164	1185	o-Xylene	0.06	0.22			0.06	0.11	0.06		0.05	0.05	0.06	0.26			0.34				A
ms10	1282	1325	Styrene				0.08			0.07	0.07		0.14		0.17		0.08	0.08	0.05	0.53	0.08	A
ms12	1310	1300	Benzene, 1,3,5-trimethyl-	0.14	0.10												0.12	0.08	0.10	0.07		A
ms18	1677	985	2,6-Octadiene, 2,6-dimethyl-		0.53			0.10	0.25	0.11			0.09								0.41	B
ms22	1936	1873	Butylated hydroxytoluene		0.54	0.38	2.27			0.55	0.82		3.31				1.35				5.31	A
Total miscellaneous				0.32	1.49	0.45	2.35	0.16	0.64	0.79	0.94	0.05	3.59	0.11	0.51	0.00	1.60	0.58	0.22	0.67	5.84	
Total volatile compounds				87.89	482.88	66.87	197.88	107.38	179.44	71.55	70.22	89.94	58.58	385.20	168.90	720.32	63.39	128.11	138.07	738.16	201.35	

^(1)^ Average of the mg/L (*n* = 3) = $\frac{\mathrm{Area}\;\mathrm{of}\;\mathrm{each}\;\mathrm{compound}\;\times \;\mathrm{Amount}\;\mathrm{of}\;\mathrm{internal}\;\mathrm{standard}\;}{\mathrm{Area}\;\mathrm{of}\;\mathrm{internal}\;\mathrm{standard}\;\times \;\mathrm{Amount}\;\mathrm{of}\;\mathrm{sample}/10^{6}\;}$; ^(2)^ Kovats indices of unknown compounds in DB-WAX column; ^(3)^ compounds by order of Kovats indices in a chemical class; ^(4)^ see code name in Table 1; ^(5)^ volatiles were identified based on the following criteria: A, mass spectrum and retention index consistent with those of an authentic standard; B, mass spectrum consistent with that of the Wiley 275 mass spectrum database.

**Table 5:** Mean sensory attribute intensity ratings^a,b^ for distilled *soju* samples determined by descriptive analysis from a panel of ten judges over duplicate replications.

Sensory Code ^A^	Sample Code ^B^																	
	JWA	JRO	MSO	HWA	IPU	R25	MBA	OAK	SRE	DJA	R40	HBI	GSO	MIR	IDO	HGO	PJU	CTO
***Aroma***																		
Alcohol_A	5.30 ^bcd^	5.95 ^abc^	5.00 ^de^	5.55 ^abcd^	4.95 ^de^	5.35 ^bcd^	5.10 ^de^	5.50 ^abcd^	5.20 ^cde^	5.10 ^de^	4.95 ^de^	5.15 ^cde^	5.45 ^abcd^	5.55 ^abcd^	4.40 ^e^	6.20 ^a^	4.75 ^de^	6.05 ^ab^
Sour_A	3.80 ^cde^	4.40 ^bcde^	5.20 ^ab^	4.45 ^bcd^	4.50 ^bcd^	5.85 ^a^	4.30 ^bcde^	3.25 ^e^	4.75 ^abcd^	4.80 ^abcd^	4.90 ^abc^	5.85 ^a^	5.00 ^ab^	5.00 ^ab^	4.55 ^bcd^	4.85 ^abc^	4.05 ^bcde^	3.65 ^de^
Sweet_A	5.95 ^bcd^	6.25 ^abc^	3.70 ^i^	6.35 ^abc^	3.90 ^ghi^	6.40 ^abc^	3.80 ^hi^	6.35 ^abc^	4.85 ^defgh^	7.20 ^a^	6.75 ^ab^	4.65 ^efghi^	4.55 ^fghi^	4.00 ^ghi^	3.85 ^ghi^	5.70 ^bcde^	4.30 ^ghi^	4.95 ^defg^
Ggrape_A	4.50 ^bcd^	4.50 ^bcd^	2.30 ^fg^	4.95 ^abc^	3.05 ^efg^	5.35 ^ab^	2.95 ^efg^	3.85 ^cde^	3.55 ^def^	5.00 ^abc^	6.10 ^a^	4.10 ^bcde^	3.55 ^def^	3.15 ^efg^	3.10 ^efg^	4.90 ^abc^	3.15 ^efg^	3.70 ^cde^
Treefr_A	4.95 ^abc^	5.45 ^ab^	2.75 ^h^	5.70 ^a^	3.40 ^efgh^	5.85 ^a^	3.30 ^efgh^	4.80 ^abcd^	4.40 ^bcde^	5.70 ^a^	5.85 ^a^	4.05 ^cdefg^	3.95 ^cdefg^	3.60 ^efgh^	4.30 ^bcdef^	5.65 ^a^	3.15 ^fgh^	3.65 ^defgh^
Pineap_A	5.25 ^b^	4.45 ^bcde^	2.20 ^j^	4.15 ^cdef^	2.40 ^j^	4.95 ^bc^	2.75 ^hij^	3.55 ^efghi^	3.15 ^fghij^	6.80 ^a^	4.65 ^bcd^	3.90 ^defg^	2.80 ^hij^	2.50 ^j^	2.50 ^j^	3.65 ^defgh^	3.10 ^ghij^	2.55 ^ij^
Barley_A	2.50 ^j^	2.80 ^hij^	5.10 ^bcd^	2.85 ^hij^	3.80 ^gf^	3.35 ^ghij^	4.65 ^def^	3.65 ^gh^	3.80 ^gf^	2.60 ^ij^	3.35 ^ghij^	4.10 ^efg^	4.90 ^cde^	5.80 ^ab^	3.80 ^gf^	2.80 ^hij^	5.60 ^abc^	3.45 ^ghi^
Yeast_A	3.00 ^hi^	3.20 ^ghi^	6.45 ^a^	3.60 ^efghi^	3.55 ^fghi^	4.25 ^defg^	4.65 ^cde^	3.50 ^fghi^	3.80 ^efgh^	2.70 ^i^	3.40 ^fghi^	4.95 ^cd^	6.05 ^ab^	5.20 ^bcd^	4.35 ^def^	3.05 ^hi^	5.70 ^abc^	3.20 ^ghi^
Earthy	2.15 ^hi^	2.20 ^hi^	6.30 ^a^	2.10 ^hi^	4.20 ^bcde^	2.90 ^fghi^	4.10 ^bcde^	3.15 ^efgh^	3.70 ^cdef^	1.90 ^i^	2.60 ^fghi^	3.65 ^cdef^	4.70 ^bc^	4.30 ^bcd^	3.10 ^efgh^	2.40 ^ghi^	5.20 ^ab^	3.40 ^defg^
Salty_A	2.60 ^jk^	2.35 ^k^	6.35 ^ab^	2.85 ^ijk^	3.35 ^ghijk^	4.05 ^fgh^	4.65 ^def^	2.85 ^ijk^	3.40 ^ghij^	2.35 ^k^	3.65 ^fghi^	5.60 ^bcd^	5.80 ^bc^	5.30 ^cde^	4.20 ^fg^	2.75 ^ijk^	5.80 ^bc^	3.15 ^hijk^
Woody_A	3.55 ^b^	4.25 ^b^	4.20 ^b^	4.45 ^b^	4.25 ^b^	4.15 ^b^	4.30 ^b^	7.00 ^a^	4.10 ^b^	3.90 ^b^	4.00 ^b^	3.60 ^b^	4.05 ^b^	3.90 ^b^	3.30 ^b^	4.40 ^b^	3.70 ^b^	7.40 ^a^
Metal_A	2.25 ^a^	2.95 ^a^	3.15 ^a^	2.40 ^a^	3.70 ^a^	2.95 ^a^	2.90 ^a^	2.90 ^a^	2.70 ^a^	2.10 ^a^	3.00 ^a^	2.90 ^a^	3.00 ^a^	3.20 ^a^	2.80 ^a^	2.80 ^a^	3.40 ^a^	3.20 ^a^
Bleach_A	3.40 ^cdef^	4.25 ^abc^	3.25 ^cdef^	2.85 ^f^	3.60 ^abcdef^	3.45 ^bcdef^	4.20 ^abcd^	4.45 ^abc^	3.65 ^abcdef^	3.50 ^abcdef^	3.60 ^abcdef^	3.60 ^abcdef^	3.50 ^abcdef^	3.00 ^def^	2.70 ^f^	4.70 ^a^	2.95 ^ef^	4.65 ^ab^
***Flavor*** **/*Taste***																		
Bitter	5.50 ^a^	6.10 ^a^	5.65 ^a^	5.25 ^a^	5.70 ^a^	5.45 ^a^	5.70 ^a^	5.80 ^a^	5.80 ^a^	5.00 ^a^	6.20 ^a^	6.25 ^a^	5.55 ^a^	5.75 ^a^	5.30 ^a^	5.25 ^a^	4.95 ^a^	5.80 ^a^
Sour	3.50 ^c^	3.55 ^c^	6.20 ^a^	3.45 ^c^	3.95 ^bc^	4.15 ^bc^	4.20 ^bc^	3.50 ^c^	3.50 ^c^	4.15 ^bc^	4.30 ^bc^	4.80 ^b^	3.75 ^bc^	4.20 ^bc^	3.95 ^bc^	4.45 ^bc^	4.05 ^bc^	3.75 ^bc^
Salty	3.05 ^i^	3.50 ^fghi^	6.25 ^a^	3.30 ^ghi^	3.85 ^defghi^	4.05 ^defg^	5.55 ^ab^	4.15 ^defg^	4.25 ^defg^	3.60 ^efghi^	4.20 ^defg^	4.60 ^bcd^	4.40 ^def^	4.60 ^bcd^	4.50 ^cde^	3.00 ^i^	4.45 ^def^	4.00 ^defgh^
Sweet	5.15 ^abc^	5.05 ^abcd^	3.95 ^ef^	5.15 ^abc^	4.35 ^cdef^	5.25 ^abc^	4.80 ^bcde^	5.40 ^abc^	4.80 ^bcde^	5.90 ^a^	5.50 ^ab^	4.40 ^cdef^	4.70 ^bcdef^	4.00 ^def^	3.70 ^f^	5.10 ^abc^	4.80 ^bcde^	4.70 ^bcdef^
Alcohol	6.10 ^a^	6.30 ^a^	5.00 ^a^	5.70 ^a^	5.85 ^a^	5.75 ^a^	5.55 ^a^	5.60 ^a^	5.80 ^a^	5.90 ^a^	6.20 ^a^	5.55 ^a^	5.75 ^a^	5.85 ^a^	5.50 ^a^	5.90 ^a^	6.00 ^a^	6.10 ^a^
Fruity	4.95 ^ab^	4.60 ^abc^	3.15 ^gf^	5.10 ^ab^	3.15 ^gf^	5.15 ^ab^	3.40 ^efg^	4.05 ^bcdef^	3.10 ^gf^	5.40 ^a^	5.35 ^a^	3.45 ^efg^	3.30 ^efg^	3.70 ^cdefg^	3.05 ^gf^	4.65 ^abc^	2.65 ^g^	3.50 ^defg^
Minty	5.05 ^a^	4.85 ^a^	3.15 ^g^	4.90 ^a^	3.95 ^bcdefg^	4.40 ^abcde^	3.65 ^efg^	4.30 ^abcde^	4.20 ^abcdef^	4.70 ^ab^	4.55 ^abcd^	3.90 b^cdefg^	3.75 ^cdefg^	3.70 ^defg^	3.40 ^fg^	4.90 ^a^	3.60 ^efg^	4.45 ^abcde^
Barley	2.95 ^ijk^	2.45 ^k^	5.65 ^ab^	3.00 ^hijk^	4.10 ^defgh^	3.70 ^efghij^	4.85 ^bcd^	4.25 ^cdefg^	4.25 ^cdefg^	3.15 ^ghijk^	3.80 ^defghij^	4.60 ^bcdef^	5.35 ^bc^	5.35 ^bc^	4.75 ^bcde^	2.80 ^jk^	5.65 ^ab^	3.50 ^fghijk^
Earthy	2.25 ^ghi^	2.20 ^hi^	5.65 ^a^	2.20 ^hi^	3.20 ^cdefghi^	2.35 ^fghi^	4.00 ^bcd^	3.00 ^defghi^	3.45 ^cdef^	2.20 ^hi^	2.80 ^efghi^	4.15 ^bc^	4.20 ^bc^	3.75 ^cde^	3.40 ^cdef^	2.15 ^i^	4.95 ^ab^	3.35 ^cdefg^
Metallic	3.15 ^bcdef^	3.30 ^bcdef^	3.70 ^abcd^	3.20 ^bcdef^	3.80 ^abc^	2.90 ^cdef^	3.65 ^abcde^	2.75 ^ef^	3.15 ^bcdef^	2.55 ^f^	2.85 ^def^	3.30 ^bcdef^	3.20 ^bcdef^	3.00 ^cdef^	3.15 ^bcdef^	3.15 ^bcdef^	3.75 ^abcd^	4.25 ^a^
***Texture*** **/*Mouthfeel***																		
Astrin	4.20 ^a^	4.00 ^a^	4.75 ^a^	4.90 ^a^	4.45 ^a^	4.45 ^a^	4.55 ^a^	5.05 ^a^	4.40 ^a^	4.40 ^a^	4.65 ^a^	5.65 ^a^	4.75 ^a^	4.45 ^a^	4.90 ^a^	3.90 ^a^	4.40 ^a^	5.25 ^a^
Cooling	4.90 ^abc^	5.00 ^ab^	3.75 ^de^	4.80 ^abc^	4.15 ^bcde^	4.60 ^abcd^	4.30 ^abcde^	4.35 ^abcde^	4.30 ^abcde^	4.25 ^abcde^	4.60 ^abcd^	3.75 ^de^	3.85 ^de^	4.10 ^bcde^	4.65 ^abcd^	5.00 ^ab^	4.05 ^cde^	5.10 ^a^
Spicy	4.35 ^a^	4.65 ^a^	4.90 ^a^	4.15 ^a^	4.95 ^a^	4.85 ^a^	4.50 ^a^	4.40 ^a^	4.95 ^a^	4.30 ^a^	5.00 ^a^	5.55 ^a^	4.50 ^a^	4.50 ^a^	4.70 ^a^	4.50 ^a^	4.20 ^a^	4.80 ^a^
Soft	5.65 ^a^	5.50 ^a^	4.80 ^a^	5.60 ^a^	4.90 ^a^	5.85 ^a^	5.15 ^a^	5.45 ^a^	5.35 ^a^	5.35 ^a^	5.25 ^a^	4.85 ^a^	5.35 ^a^	4.90 ^a^	4.85 ^a^	5.60 ^a^	5.05 ^a^	5.70 ^a^
Body	4.35 ^cdef^	4.45 ^bcdef^	5.45 ^ab^	3.80 ^f^	4.25 ^def^	4.55 ^bcdef^	4.50 ^bcdef^	5.35 ^abc^	4.60 ^abcdef^	3.95 ^ef^	5.35 ^abc^	5.10 ^abcd^	4.70 ^abcdef^	4.60 ^abcdef^	4.75 ^abcdef^	4.45 ^bcdef^	5.00 ^abcde^	5.65 ^a^

^a–k^ Mean values with the same letter in a row are not significantly different, with significance set at *p* < 0.05 by Fisher’s least significant difference test. The intensity of the attributes ranged from 0 to 9 (0, none; 1, very weak, 5: moderate, 9: very strong). ^A,B^ The sensory attribute codes and samples are defined in Table 1 and Table 2.

**Table 6:** Coordinates, factor contributions (%), and squared cosine values of the groups of variables by MFA.

	Coordinates		Contributions (%)		Squared Cosines	
Active Data Sets	F1	F2	F1	F2	F1	F2
Chemical	0.84	0.20	34.00	25.60	0.61	0.03
Sensory	0.76	0.25	30.89	31.87	0.53	0.057
Volatile	0.86	0.33	35.10	42.51	0.46	0.069

## Data Availability

Not applicable.

## Sample Availability

Samples of the distilled *soju* are available from the authors.
